#  Corrigendum

**DOI:** 10.1002/jcla.24523

**Published:** 2022-05-31

In Volume 34, Issue 12 (2020), Figure 1 was incorrect in the article titled “Promotive effect of Talin‐1 protein on gastric cancer progression through PTK2‐PXN‐VCL‐E‐Cadherin‐CAPN2‐MAPK1 signaling axis” by Hongzhu Yan, Min Guo, Jue Zou, Feng Xiao, Lina Yi, Ying He, Bosheng He.

Incorrect Figure 1


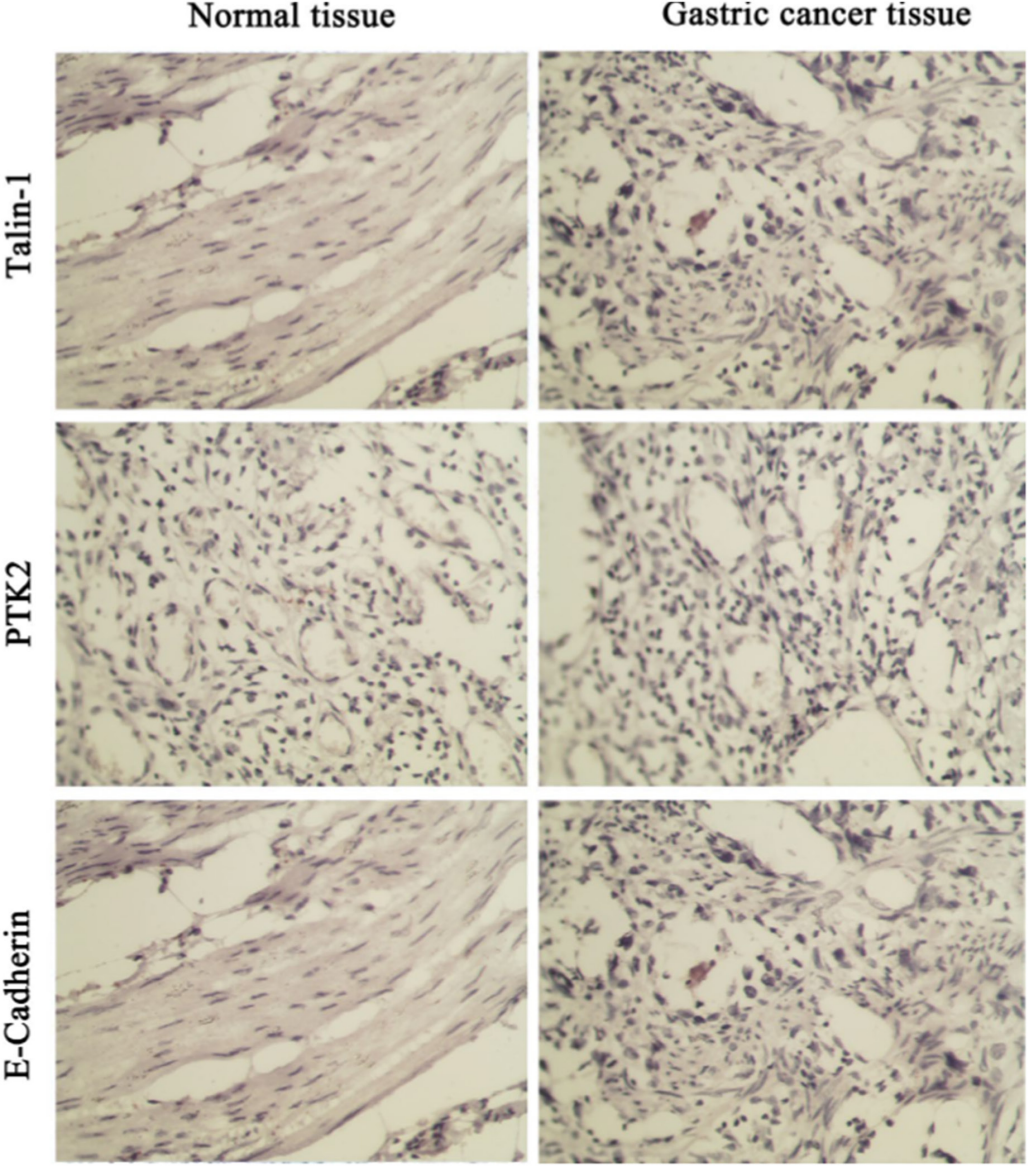


Correct Figure 1


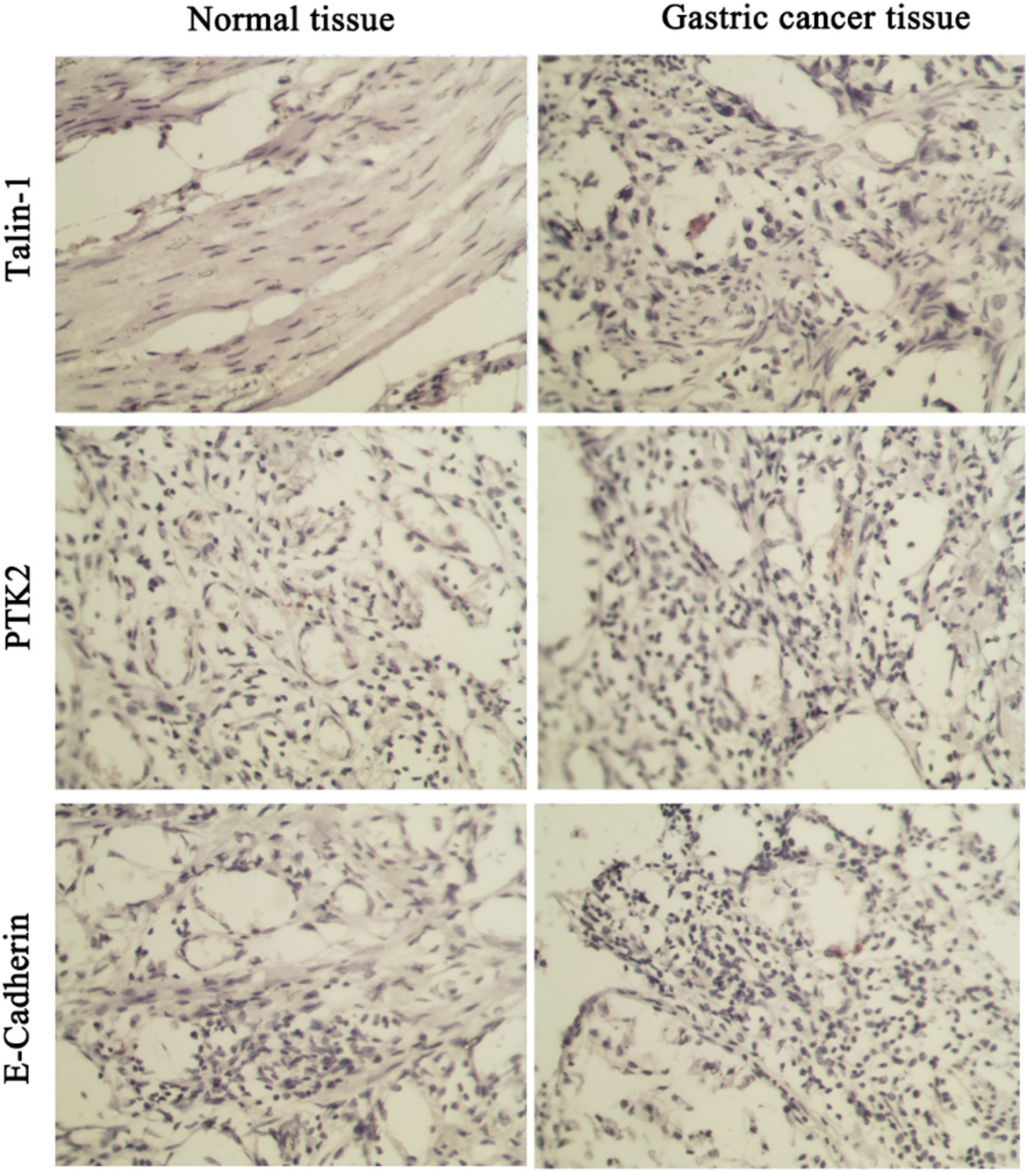


The authors regret the preparation error and apologize for any confusion.

